# Processing of Spoken Emotions in Schizophrenia: Forensic and Non-forensic Patients Differ in Emotional Identification and Integration but Not in Selective Attention

**DOI:** 10.3389/fpsyt.2022.847455

**Published:** 2022-03-21

**Authors:** Rotem Leshem, Michal Icht, Boaz M. Ben-David

**Affiliations:** ^1^Department of Criminology, Bar-Ilan University, Ramat Gan, Israel; ^2^Department of Communication Disorders, Ariel University, Ariel, Israel; ^3^Baruch Ivcher School of Psychology, Reichman University (IDC), Herzliya, Israel; ^4^Department of Speech-Language Pathology, University of Toronto, Toronto, ON, Canada; ^5^Toronto Rehabilitation Institute, University Health Networks, Toronto, ON, Canada

**Keywords:** schizophrenia, emotions, processing of emotions, speech processing, cognition, selective attention, forensic psychiatry

## Abstract

Patients with schizophrenia (PwS) typically demonstrate deficits in visual processing of emotions. Less is known about auditory processing of spoken-emotions, as conveyed by the prosodic (tone) and semantics (words) channels. In a previous study, forensic PwS (who committed violent offenses) identified spoken-emotions and integrated the emotional information from both channels similarly to controls. However, their performance indicated larger failures of selective-attention, and lower discrimination between spoken-emotions, than controls. Given that forensic schizophrenia represents a special subgroup, the current study compared forensic and non-forensic PwS. Forty-five PwS listened to sentences conveying four basic emotions presented in semantic or prosodic channels, in different combinations. They were asked to rate how much they agreed that the sentences conveyed a predefined emotion, focusing on one channel or on the sentence as a whole. Their performance was compared to that of 21 forensic PwS (previous study). The two groups did not differ in selective-attention. However, better emotional identification and discrimination, as well as better channel integration were found for the forensic PwS. Results have several clinical implications: difficulties in spoken-emotions processing might not necessarily relate to schizophrenia; attentional deficits might not be a risk factor for aggression in schizophrenia; and forensic schizophrenia might have unique characteristics as related to spoken-emotions processing (motivation, stimulation).

## Introduction

Schizophrenia involves a range of cognitive, behavioral, and emotional dysfunctions. The clinical picture includes both positive symptoms, such as hallucinations or delusions, and negative symptoms, such as emotional and social withdrawal. Prominent among the negative symptoms are diminished emotional expressions and identification, as related to body language and intonation of speech (prosody) (1). These affective disturbances may lead to significant problems in social interactions and communication. In turn, difficulties in processing spoken emotions may present a risk factor for maladaptive behaviors (2, 3).

Most of the literature on emotional processing in schizophrenia focuses on the visual modality, including emotional images and facial expressions (4). For example, patients with schizophrenia (PwS) have been found to misattribute emotions to neutral facial expressions, as well as misinterpret emotional facial expressions (5). These misapprehensions can be related to impaired adaptive behavior in daily life situations (6). Much less is known about emotional processing in the auditory modality using spoken stimuli in this population (7). Spoken emotional processing involves the correct identification and integration of information conveyed by two auditory channels, semantics (words) and prosody (tone of speech) (8). The literature suggests that emotional processing in these two channels may be impaired in schizophrenia. Abnormal *semantic* processing and representations were found in this population, such as impaired semantic judgment (9, 10) and semantic priming (11–13). There is also evidence pointing to difficulties in identification of emotional *prosody* in schizophrenia, which may be related to deficits in basic auditory processing (14–16).

Correct spoken emotional processing often calls for selective-attention, as listeners attempt to focus on one dimension (e.g., prosody) while actively ignoring the information presented in the other dimension (semantics) (8). Indeed, deficits in selective-attention and inhibition are typical of schizophrenia. They have been identified in the visual domain (e.g., color-word Stroop) (17–19), with cross-modal visual-auditory stimuli [for a review, see (4)], and in the auditory domain (11, 13). In other daily situations, speech processing may involve the integration of the two channels to generate a coherent spoken message. Deficits in sensory information integration, that are also common in schizophrenia (20–22), might impair this ability as well.

A recent study conducted in our lab (23) examined emotional processing in forensic PwS who committed severe violent offenses, as compared to their non-clinical peers (controls). Three main trends of spoken emotion processing were indicated. (A) Identification: forensic PwS were able to identify spoken emotions, yet their emotional-discriminability was poorer than that of controls; (B) Selective-attention: forensic PwS' performance indicated larger failures of selective-attention than their peers; and (C) Integration: forensic PwS integrated the prosodic and semantic channels in the same fashion as controls. Namely, both groups similarly gave prominence to the prosodic information over the semantic one (prosodic dominance), a marker of typical spoken-emotion processing, as found already in various studies (24–28).

Overall, these similarities in spoken-emotion processing between forensic PwS and controls may appear to be at odds with the pertinent literature. However, one must note that this sub-group is not fully representative of the schizophrenia population. Indeed, <10% of PwS are also violent offenders (29, 30). The literature suggests that forensic schizophrenia may be associated with unique characteristics of emotional processing, relative to non-forensic schizophrenia. However, only a limited number of studies have directly compared these PwS groups on emotional processing tasks, mainly in the visual domain. These generally show that forensic PwS may outperform non-forensic PwS (and even healthy controls) in emotional identification (31–34). Notwithstanding other studies pointing at similar or worse performance for forensic PwS (35, 36).

The emotional and mental profile of forensic PwS, in particular the increased sensitivity to emotions and heightened arousal, has been suggested to be at the root of their violent behavior (36, 37). Moreover, forensic PwS were found to show enhanced cognitive mentalizing abilities relative to non-forensic PwS (37, 38), as well as better ability to infer cognitive-mental states in others [second order theory of mind, (39)]. These abilities were associated with premeditated violent behaviors (37). Also, violent behavior may be caused at least in part by a failure or reduced ability to regulate emotions (40, 41), after they been correctly identified. Namely, forensic PwS' reduced ability to regulate emotions after they have been identified, to accurately assess the intensity of the emotional stimuli (34), and to empathize with the speaker (39) may lead to violent behavior.

In light of the differences between forensic and non-forensic PwS, the goal of the current study was to assess the processing of spoken-emotions in non-forensic schizophrenia, and compare it to our previous findings with forensic PwS (23). We used the same paradigm [T-RES, 6] and stimuli as employed in Leshem et al. (23), with a group of PwS without a history of violent behavior. In three separate tasks, participants were presented with spoken sentences in which the emotional semantic and prosodic content appeared in various combinations of congruency and incongruency from trial to trial. Participants were asked to rate the extent to which a predefined emotion was expressed by the prosody alone (prosodic-rating task), the semantics alone (semantic-rating task), or the spoken sentence as a whole (general rating task). The performance of non-forensic PwS in the current study was compared to that of forensic PwS in our previous study.

In general, the schizophrenia literature suggests difficulties in identification of spoken- emotions (4). We expected the performance of non-forensic PwS in the current study to reflect these impairments. However, Leshem et al. (23) found some preserved emotional-processing aspects for forensic PwS. Thus, we expected significant differences between the two schizophrenia groups. The following specific predictions were made: (A) Identification: we expected the non-forensic group to be more impaired than the forensic group in emotion discriminability; (B) Selective-attention: failures of selective-attention were expected to be similar, as both groups share a core cognitive profile; (C) Integration: reduced prosodic dominance was expected for the non-forensic group, as compared to the forensic group.

## Materials and Methods

The study received ethics approval from the medical center and the academic institute affiliated with the first author. The study was carried out in accordance with the Declaration of Helsinki, and informed consent was obtained from all individual participants.

### Participants

The non-forensic schizophrenia group consisted of 45 male participants diagnosed with schizophrenia, who volunteered to participate with no monetary compensation. All participants were recruited from community-based programs, such as hostels and sheltered housing. All participants received mental-health support from the public health system.

These participants were compared to a group of forensic schizophrenia and their controls taken from Lesehm et al. (23). Note, data for the current study was collected in tandem to data collection in Leshem et al., by a different research team (based in the first author's academic institute). Thus, the studies were conducted without knowledge of each other's results.

The forensic schizophrenia group consisted of 21 male participants diagnosed with schizophrenia with a violent criminal record, who volunteered to participate with no monetary compensation, recruited from the Maximum Secure Unit (MSU). All were under court-ordered compulsory hospitalization due to severe violent behaviors (including murder and rape). The control group consisted of 21 male volunteers from the general population that matched the forensic group in socio-demographic characteristics. They were recruited by advertisements in and around the campus (including a local mall) and received the equivalent of $25 to compensate for their participation time.

*Inclusion criteria for the clinical groups*. (1) normal hearing (with no reported pathologies or history of hearing disorders), normal or corrected-to-normal vision, based on self-report (42); (2) a diagnosis of schizophrenia (ICD-10 schizophrenia) (43), ≥1 year from initial diagnosis; (3) no indication of change to their treatment regimen during the last 4 months, as per medical records; (4) no history of substance addiction in the year prior to the study, as per medical records; (5) no history of head trauma, or neurological illness, as per medical records and/or staff reports; (6) possessed the capacity to provide informed consent, with intellectual abilities within the normal range (as evaluated by the clinical staff); (7) age range 20-55 yrs.

*Participants' characteristics*. The non-forensic, forensic and control groups did not differ in mean age, *M* = 38.2 yrs (*SD* = 9. 8), M = 36.3yrs (*SD* = 9.3), and *M* = 34.3yrs (*SD* = 9.3) respectively, *t*_(64)_ = 0.75, *p* = 0.45 and *t*_(64)_ = 1.6, *p* = 0.10; or in years of education *M* = 11.6 yrs (*SD* = 1.7), *M* = 11.5yrs (*SD* = 2.4) and *M* = 12.1yrs (*SD* = 0.7), respectively, *t*_(64)_ = 0.28, *p* = 0.78 and *t*_(64)_ = 1.35, *p* = 0.18. However, the percentage of native Hebrew speakers was higher for the non-forensic group, 82%, than for the forensic group, 52%, $\chi_{(}^{2}{}_{1)}$ = 6.43, *p* = 0.011, and the control group, 57%, $\chi_{(}^{2}{}_{1)}$ = 4.7, *p* = 0.03. Therefore, we controlled for this factor in all following analyses.

### Measures and Tools: Test of Rating of Emotions in Speech

The Hebrew version of the T-RES (44) was used, with the following emotions: anger, happiness, sadness, and baseline neutrality. The T-RES consists of three tasks. Two relate to selective-attention and identification: (a) prosodic-rating and (b) semantic-rating, in which listeners are requested to rate the emotion based only on prosodic/semantic information, respectively. The third task was general rating, an integration task in which listeners are requested to rate the emotion of the sentence as a whole. All spoken sentence stimuli had been pre-recorded by a professional female actress.

#### Stimuli

**Figure 1:**
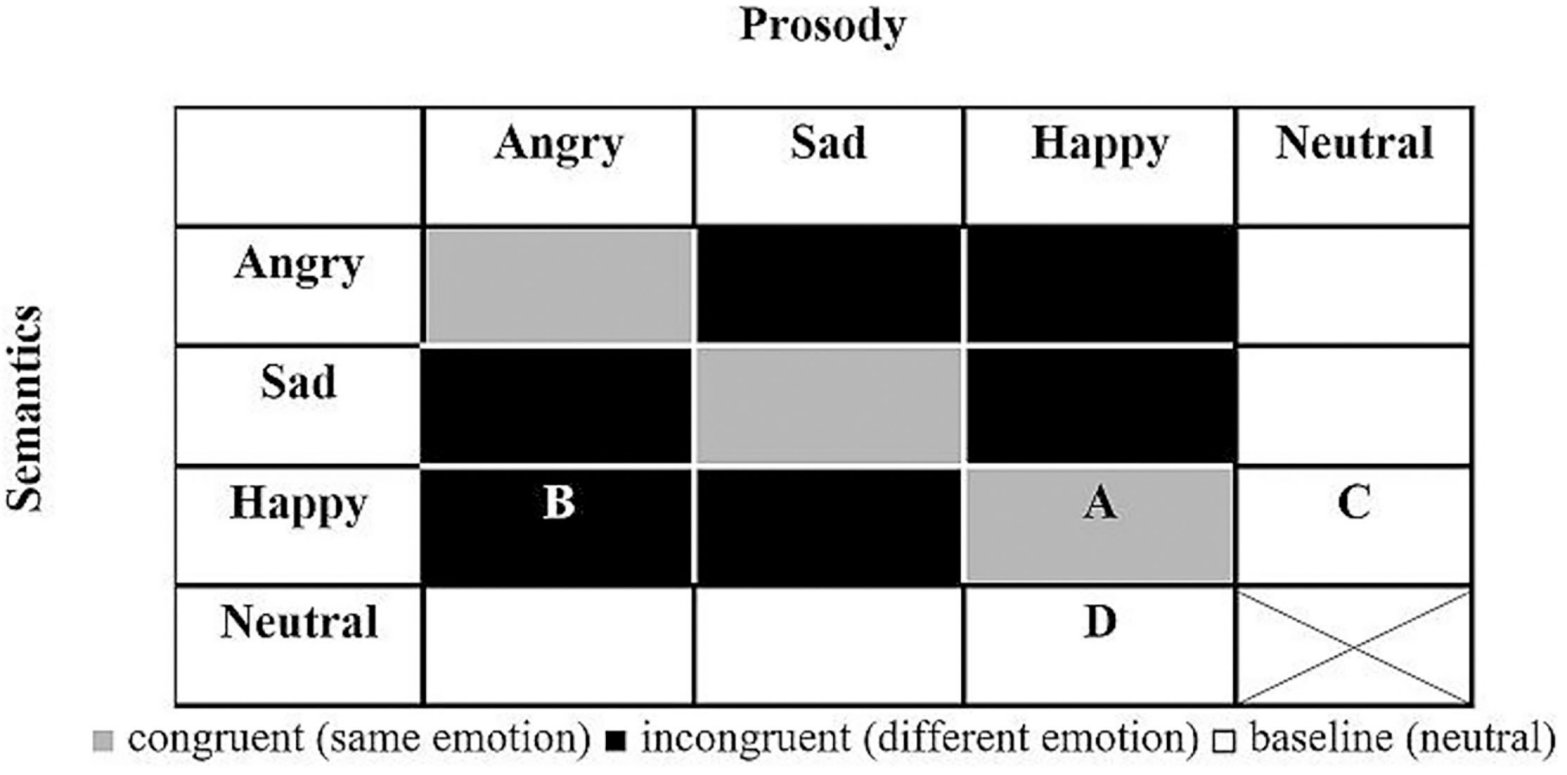
General design of T-RES stimuli. All combinations of prosody and semantics (15) are presented in each emotional rating block (neutral semantics spoken with neutral prosody was deemed uninformative and confusing and was not presented). A, example of congruent stimulus (happy semantics and happy prosody); B, example of incongruent stimulus (happy semantics and angry prosody); C, example of baseline semantics (happy semantics and neutral prosody); D, example of baseline prosody (neutral semantics and happy prosody).

Figure 1 presents the makeup of the T-RES stimuli: the 15 spoken sentences in each semantic category are represented once in each of the tested prosodies, generating a 4 (semantic) × 4 (prosody) matrix. For a full description of the characteristics of the spoken sentences and how they were constructed, see Ben-David et al. (25, 26, 45, 46).

#### Reliability, Sensitivity, and Validity

We used the Hebrew version of the T-RES. Content validity (47) has been confirmed by verifying that all sentences are distinctive and exemplars of their respective lexical categories [for method, see (45)] and prosodic categories (46). The T-RES was also found to be valid and sensitive to detect population-related differences in various studies. For example, expected aging-related differences in selective attention, as suggested from the pertinent literature, were confirmed by comparing T-RES performance for younger and old adults (25). Similarly, differences as related to tinnitus and cochlear implant usage were also detected by the T-RES, further supporting its validity and sensitivity (28, 48). Recently, the T-RES' reliability was confirmed as data for young adult undergraduates were found to be equivalent across studies and platforms (27).

#### Apparatus

The spoken sentences were presented on a 2.20 GHz Intel personal computer, using a 15.4-in. LCD monitor, via professional AKG K240 headphones, at a comfortable listening level [as confirmed by each participant, see (49)]. A research assistant was present throughout the experimental session, which lasted about 30 min.

### Procedure

Upon arrival, all participants received an explanation of the experimental tasks and those wishing to participate signed an informed consent form. The T-RES session was conducted only after participants were found to meet the inclusion/exclusion criteria. Subsequently, all participants were tested individually in a quiet room at their place of residence (i.e., hostel or sheltered housing).

In the T-RES, each sentence is rated on three separate rating blocks. For each trial, using a six-point Likert scale, listeners are asked to rate “How much do you agree that the speaker conveys______ (anger, sadness, or happiness)? From 1—strongly disagree to 6—strongly agree.”

The experimental session began with the general rating task. For half of the participants, this was followed by the semantic-rating task and then the prosodic-rating task; for the other half, this order was reversed. The order of the three emotion-rating blocks was counterbalanced by using the Latin square design, and the order of the trials in each block was fully randomized. In sum, each sentence was presented three times in each task, once in each of three rating blocks (anger, sadness, and happiness), with a total of 135 trials per session. The full description of the T-RES design is specified in previous works (25, 26).

### Statistical Analyses

All analyses used mixed linear modeling, MLM (SPSS Statistics 28), with average ratings as the dependent variable, Group (x2: forensic vs. non-forensic PwS) and Native Language (x2: native Hebrew speaker or not) as between participant variables. The goal of each analysis was to conduct a 2-way interaction of Group and the test-specific variable (Emotion Identification, Selective-attention, or Prosodic Dominance). To control for possible biases, the following fixed effects were included: Group, Target Emotion (x3: anger, sadness, or happiness) and Native Language and their interactions with the test-specific variable. Across analyses, averages represent MLM estimates for the data collected in the current study and Leshem et al. (23). In Appendix A, we add another equivalent analysis, comparing the novel non-forensic PwS group with the control group recruited for Leshem et al. (23). Note, MLM analysis was used as it is more robust than general linear models (ANOVA), specifically when testing unequal groups (50), and does not require normal distribution of errors, sphericity and other assumptions necessary for the ANOVA analysis.

## Results

### Identifications of Emotions Presented in the Prosodic and Semantic Channels

**Figure 2:**
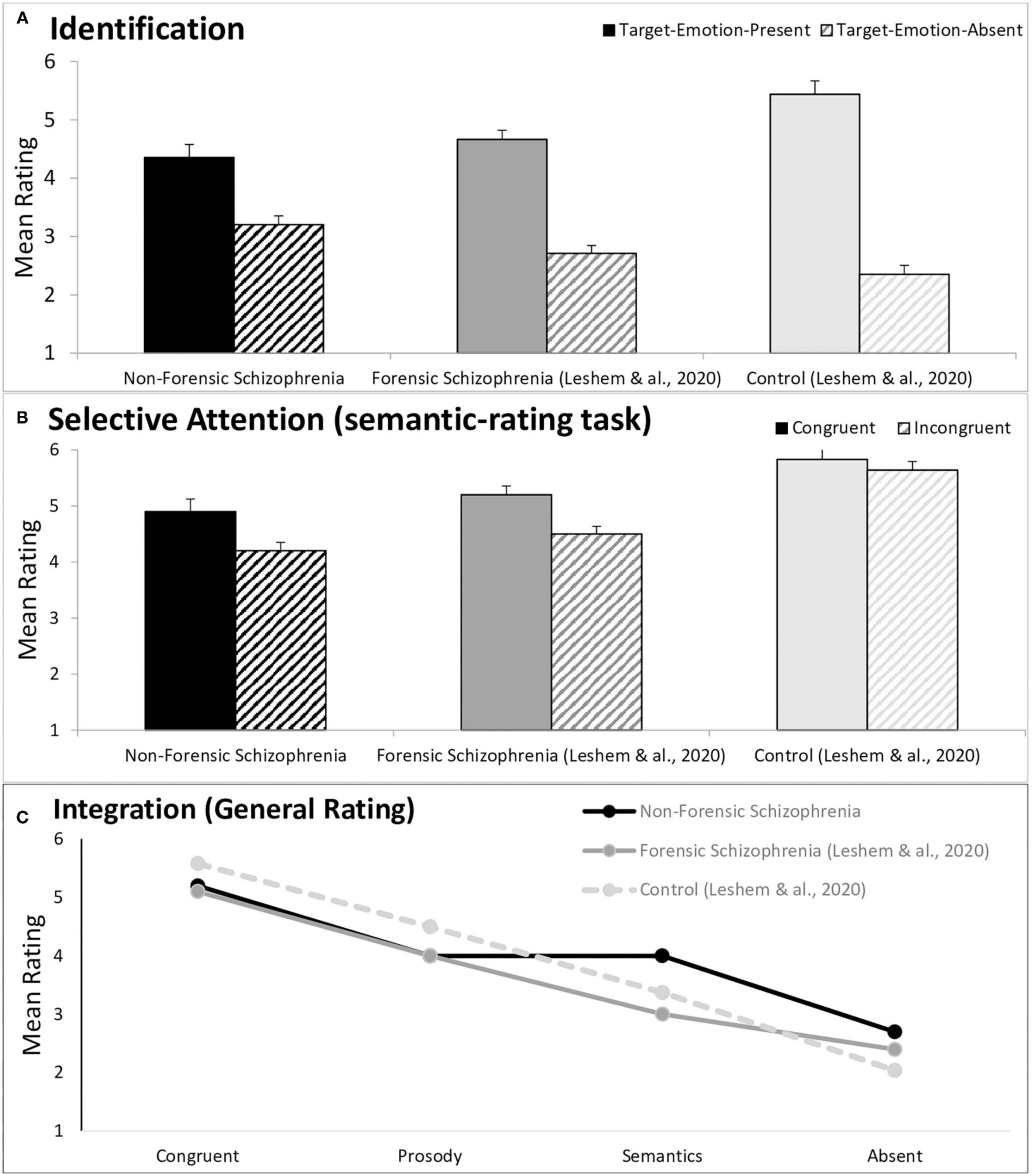
A graphic description of ratings in the T-RES tasks, separately for non-forensic PwS (black bars, data from the current study), forensic PwS [gray bars, taken from Leshem et al. (23)] and Controls [light gray bars, Leshem et al. (23)]. All data are estimates of MLM models averaged across the three emotion rating blocks. The error bars represent standard errors. **(A)** Identification, comparing target emotion-present and target-emotion-absent trials, averaged across the prosodic- and semantic-rating tasks; **(B)** Selective-attention, comparing congruent and incongruent trails, in the semantic-rating task; **(C)** Integration, presenting target-emotion-congruent, -prosody, -semantics and -absent trials.

The extent of Emotion-Identification was gauged by comparing average ratings of target-emotion-present and target-emotion-absent trials in baseline sentences, when the to-be-ignored channel was neutral (white cells in Figure 1). Target-emotion-present trials are sentences that present the target emotion in the to-be-rated channel (thus, a high rating is expected), while in target-emotion-absent trials, a different emotion is presented in the to-be-rated channel (thus, a low rating is expected). A higher Emotion-Identification score suggests better discrimination between emotions; whereas a score of 0 suggests an inability to identify the target emotion. The data is presented in the upper section of **Table 2**, and graphically displayed in Figure 2A. The aforementioned MLM analysis was conducted.

The full tests of fixed effects are available in **Table 2**. Mainly, we found a significant main effect for Emotion-Identification, *F*_(1, 64.1)_ = 117.1, *p* < 0.001, and a significant interaction of Emotion-Identification ^*^ Group, *F*_(2, 63.4)_ = 4.25, *p* = 0.012. These effects indicate that the extent of Emotion-Identification was larger for the forensic than for the non-forensic group. However, planned comparisons suggest that the effect of Emotion-Identification was significant in each group separately, *F*_(1, 64.8)_ = 78.6, *p* < 0.001, and *F*_(1, 64.8)_ = 42.1, *p* < 0.001, for the forensic and non-forensic groups, respectively. No triple interaction was found for Target-Channel ^*^Emotion-Identification ^*^ Group, *F* < 1, indicating that the group difference in Emotion-Identification was the same across both acoustic channels.

To conclude, both groups clearly identified the presented emotions in both prosody and semantics. However, in line with our first prediction, the forensic group's ratings indicate significantly better discrimination between emotions than the non-forensic group. Recall that the Leshem et al. (23) data and analyses indicated better emotional discrimination (as indicated by higher Emotion-Identification scores) for matched controls than for forensic PwS. Taken together, the following gradient of Emotion-Identification is observed: non-forensic PwS (M = 1.2) < forensic PwS (M = 2.0) < controls (M = 3.1), as graphically presented in Appendix B. Analysis presented in Appendix A confirms that Emotion-Identification for non-forensic PwS was significantly smaller than for controls [taken from Lesham et al. (23)], *F*_(1, 63.8)_ = 44.7, *p* < 0.001.

### Selective-Attention to the Prosodic or the Semantic Channel

Selective-attention was gauged by comparing average ratings of congruent sentences (presenting the rated-emotion *in both channels*) with ratings of incongruent sentences (presenting the rated-emotion *in the target channel*, and a different emotion in the other channel). Higher selective-attention scores indicated larger failures of selective-attention; a score of 0 suggests no such failures occurred. The data are presented in midsection of Table 1 and graphically displayed in Figure 2B.

**Table 1:** Summary of ratings (Means and standard errors), averaged across target emotions (estimates of the MLM model), for the forensic patients with schizophrenia and the non-forensic patients with schizophrenia, with *F* values of the group comparison.

	**Forensic**		**Non-forensic**		**Group effects**
	**Prosody**	**Semantic**	**Prosody**	**Semantic**	
**Identification (baseline sentences)**					
Target-emotion-present	4.7 (0.02)	4.7 (0.24)	4.5 (0.16)	4.2 (0.18)	
Target-emotion-absent	2.7 (0.18)	2.7 (0.15)	3.3 (0.14)	3.2 (0.12)	
**Emotion-identification score (target-emotion-present vs. target-emotion-absent), averaged across channels**					
	2.0 (0.22)		1.1 (0.18)		*F*_(1, 63)_ = 4.73, *p* = 0.012
**Selective-attention**					
Congruent	4.9 (0.19)	5.2 (0.18)	4.9 (0.15)	4.9 (0.14)	
Incongruent	4.7 (0.17)	4.5 (0.21)	4.5 (0.14)	4.2 (0.16)	
**Selective-attention failure score (congruent vs. incongruent), averaged across channels**					
	0.5 (0.12)		0.5 (0.15)		*F*_(1, 63.8)_ = 0.81, *p* = 0.45
**Integration**					
Congruent sentences	5.1 (0.14)		5.2 (0.10)		*F*_(1, 162.3)_ = 0.38, *p* = 0.54
Prosodic sentences	4.0 (0.14)		4.0 (0.10)		*F*_(1, 184.6)_ = 0.0, *p* = 0.99
Semantic sentences	3.0 (0.15)		4.0 (0.11)		*F*_(1, 187.6)_ = 24.5 *p* <0.001
Target-emotion-absent	2.4 (0.13)		2.7 (0.10)		*F*_(1, 192.2)_ = 5.8, *p* = 0.017
**Prosodic-dominance score (prosodic vs. semantic)**					
	1.0 (0.26)		0.0 (0.21)		*F*_(1, 63)_ = 8.38, *p* = 0.001

The full tests of fixed effects are available in Table 2. Mainly, a significant effect for Selective-Attention was found, *F*_(1, 67.6)_ = 22.3, *p* < 0.001, that did not interact with Group, *F* < 1. A significant interaction of Target-Emotion ^*^ Selective-Attention was found, *F*_(2, 66)_ = 3.97, *p* = 0.024, indicating larger failures of selective-attention when listeners were asked to focus on the semantics and ignore the prosody, than vice versa (Selective-Attention scores: 0.69 vs. 0.27, respectively). But most importantly, no triple interaction of Group ^*^ Selective-Attention ^*^ Target-Channel was found, *F* < 1, suggesting that the impact of Target-Channel on Selective-Attention did not differ between groups.

**Table 2:** The full MLM analyses.

**Emotional identification**		**Selective-attention**		**Integration: prosodic-dominance**	
Intercept	*F*_(1, 64.8)_ = 3056.4, *p* <0.001	Intercept	*F*_(1, 66.6)_ = 2689.8, *p < * 0.001	Intercept	*F*_(1, 64.1)_ = 1900.56, *p < * 0.001
Group	*F*_(1, 63.5)_ = 0.5, *p* = 0.48	Group	*F*_(1, 63)_ = 1.36, *p =* 0.25	Group	*F*_(1, 63.0)_ = 12.85, *p =* 0.001
Native-language	*F*_(1, 63)_ = 0.2, *p* = 0.7	Native-language	*F*_(1, 63)_ = 0.02, *p =* 0.9	Native-Language	*F*_(1, 63.0)_ = 0.81, *p =* 0.37
Target-emotion	*F*_(2, 65)_ = 7.8, *p* = 0.001	Target-Emotion	*F*_(2, 65)_ = 1.6, *p =* 0.21	Target-Emotion	*F*_(2, 65.0)_ = 20.71, *p < * 0.001
Target-channel	*F*_(1, 66.6)_ = 1.0, *p =* 0.32	Target-channel	*F*_(1, 65)_ = 0.31, *p =* 0.57		
Emotional-identification	*F*_(1, 64.1)_ = 117.1, *p < * 0.001	Selective-attention	*F*_(1, 67.6)_ = 22.27, *p < * 0.001	Prosodic-dominance	*F*_(1, 64.6)_ = 7.69, *p =* 0.007
Emotional-identification* native-language	*F*_(1, 63)_ = 11.16, *p =* 0.001	Selective-attention* native-language	*F*_(1, 63)_ = 0.13, *p =* 0.72	Prosodic-dominance* Native Language	*F*_(1, 63.0)_ = 3.87, *p =* 0.053
Emotional-identification* target-emotion	*F*_(2, 65)_ = 16.33, *p < * 0.001	Selective-attention* target-emotion	*F*_(2, 65)_ = 3.67, *p =* 0.03	Prosodic-dominance* Target-Emotion	*F*_(2, 65.0)_ = 25.49, *p < * 0.001
Emotional-identification* Target-channel	*F*_(1, 64.5)_ = 0.23, *p =* 0.64	Selective-attention* target-channel	*F*_(1, 65.4)_ = 7.83, *p =* 0.007		
Emotional-Identification* Group	*F*_(2, 63.4)_ = 8.29, *p =* 0.005	Selective-attention* Group*	*F*_(1, 64.6)_ = 0.151, *p =* 0.70	Prosodic-dominance X Group	*F*_(1, 63.0)_ = 9.24, *p =* 0.003
Emotional-Identification* Group* Target-channel	*F*_(2, 64)_ = 0.54, *p =* 0.59	Selective-attention* Group* target-channel	*F*_(2, 64)_ = 1.38, *p =* 0.26		

To conclude, in accordance with our second prediction, it appears that failures of selective-attention were significant for both groups, and were not affected by group membership. Taken with the data and analyses from Leshem et al. (23) that indicated impaired selective-attention for forensic PwS as compared to controls, the following gradient is observed: non-forensic PwS (M = 0.7) = forensic PwS (M = 0.7) < controls (M = 0.2; for the semantic-rating task, as graphically presented in Appendix B). Analysis presented in Appendix A confirms that the extent of Selective-Attention failures for the current non-forensic PwS group was significantly smaller than for controls [taken from Lesham et al. (23)], *F*_(1, 63.8)_ = 44.7, *p* < 0.001.

### Integration of Channels and Channel Dominance

The data are presented in the lower section of Table 1 and graphically displayed in Figure 2C. From left-to-right, Figure 2C presents average ratings for congruent trials (the rated emotion appears in both channels), prosody trials (the rated emotion appears only in the prosody), semantic trials (the rated emotion appears only in the semantics), and target-emotion-absent trials (the rated emotion is absent from prosody and semantics).

As a first step, we tested the Prosodic Dominance, gauged by comparing average ratings of prosody trials and semantic trials. A larger Prosodic Dominance (denoting a larger difference) suggests that listeners assign higher weights for the prosodic than for the semantic channel; a score of 0 suggests similar weights assigned for the two channels.

The full analysis is presented in Table 2. Mainly, analyses showed that the significant main effect of Prosodic Dominance, *F*_(1, 64.6)_ = 7.7, *p* = 0.007, significantly interacted with Group, *F*_(1, 63.0)_ = 9.2, *p* = 0.003. Planned comparisons indicated that the significant Prosodic Dominance for the forensic group, *F*_(1, 65.1)_ = 13.97, *p* < 0.001 (with a mean difference of 1.0) was not evident for the non-forensic group, *F*_(1, 65.3)_ = 0.01, *p* = 0.92 (with a mean difference of 0).

In the second step, we conducted independent paired-comparisons for each of the Types of Trials. Analyses showed that the non-forensic group provided significantly higher ratings than the forensic group for the semantics trials, *F*_(1, 187.6)_ = 24.6 *p* < 0.001, and for the target-emotion absent trials, *F*_(1, 192.2)_ = 5.9, *p* = 0.016. However, no significant group differences were noted for the congruent trials nor for the prosodic trials, *F* < 1. The tests failed to show significant interactions for Target-Emotion ^*^ Group, nor for Prosodic-Dominance ^*^ Target-Emotion ^*^ Group, *F* < 1 for all. These results suggest that differences between the two PwS groups were not dependent on the target-emotion.

In sum, our third prediction was confirmed. The forensic group assigned significantly higher weights for the prosodic than for the semantic information—significant prosodic dominance. However, this effect completely disappeared for the non-forensic group, as their semantic ratings were higher than those of the forensic group. Taken with the data and analyses from Leshem et al. (23) that indicated similar prosodic dominance for forensic PwS and their matched controls, the following gradient is observed (graphically displayed in Appendix B): non-forensic PwS (M = 0) < forensic PwS (M = 1.0) ≈ controls (M = 1.1). Analysis confirms that prosodic dominance is significantly smaller for the current non-forensic PwS group than for controls [taken from Szycik et al. (21)], *F*_(1, 63.0)_ = 12.94, *p* = 0.001.

In addition, higher ratings of target-emotion-absent trials suggest some form of decreased emotional discrimination for the non-forensic relative to the forensic group. Leshem et al's data indicated that discrimination for the forensic group was significantly worse than that for the control group, suggesting a gradient in emotional discrimination: non-forensic Pws < forensic PwS < controls (target-emotion-absent scores, 2.7, 2.4, 2.0, respectively). Again, a separate analysis confirms the difference between the non-forensic PwS group and controls [taken from Szycik et al. (21)], *F*_(1, 190.6)_ = 21.12, *p* < 0.001.

## Discussion

The current study tested processing of spoken emotions for 45 non-forensic PwS and compared their performance with that of 21 forensic PwS and their 21 controls, as obtained in our previous study (23). Forensic PwS form a unique subgroup of PwS with specific emotional abilities (e.g., improved mentalization). A few studies directly compared forensic and non-forensic PwS on emotional processing in the visual domain with mixed evidence. The current study is the first to compare the two on the auditory domain. The following trends were found: (A) Identification. Both groups were able to identify spoken emotions. However, the forensic group performance indicated better emotion-discrimination than that of the non-forensic group; (B) Selective-attention. Failures of selective-attention were indicated in both groups to a similar extent; (C) Integration. The two groups significantly differed in their ratings. The forensic group's ratings indicated a prosodic dominance, a marker for integration of emotional channels in non-clinical population. In contrast, the non-forensic group failed to show this pattern, with equal weights assigned for the prosodic and semantic information; (D) Control. In comparison to the control group, non-forensic PwS' performance on all three aspects of emotional processing was impaired.

### Identifications and Discrimination of Emotions

In accordance with our first research prediction, the non-forensic group performed with poorer discrimination of emotions than the forensic group. This pattern was evident by: (1) lower Emotion-Identification scores in both semantic-ratings and prosodic-ratings for the non-forensic group; and (2) higher ratings for target-emotion-absent trials in the general-rating task for the non-forensic group. Note that in our previous study, forensic PwS performed with poorer emotion discrimination than their non-clinical peers. Indeed, our analysis found that emotion identification for non-forensic PwS is substantially poorer than that of non-clinical population. These findings correspond with the literature on schizophrenia (3, 16, 51). Indeed, PwS are characterized by emotional dysregulation. This may manifest in both emotional expression, such as laughing in the absence of an appropriate stimulus (1), and in emotional processing, such as identifying the existence of an emotion even though it is absent from the input information (as found in the current study).

The advantage in emotional discrimination for forensic over non-forensic PwS closely follows findings by Silver et al. (34). It may suggest that reduced emotional discrimination of incoming information (associated with negative symptoms of schizophrenia) is present to a larger extent in non-forensic than in forensic schizophrenia. This ability, which is affected by schizophrenia to a lesser degree in the forensic group (as compared to the non-forensic group) may lead to higher stimulation by emotional cues, which in turn may contribute to the tendency of forensic PwS for violent behavior, especially in confrontational situations. In a similar vein, for the non-forensic PwS, severely reduced emotional discrimination may reduce emotional stimulation, explaining their behavioral withdrawal from social interactions, and the reduced risk of violent behavior.

### Selective-Attention

Validating our second research prediction, the non-forensic and the forensic groups did not differ in the extent of selective-attention failures, as indicated in both semantic-rating and prosodic-rating tasks. For example, when asked to focus only on the emotional semantic content of a spoken sentence, listeners in both groups were affected by the emotional prosodic content to the same extent. Interestingly, analyses show that selective-attention is poorer for both the forensic PwS and the non-forensic PwS groups than for non-clinical controls. Taken together, attentional abilities appear to be similarly impaired in the two groups of PwS. These findings echo the literature, demonstrating that schizophrenia is characterized by a general reduction of cognitive functions, and specifically attentional control (52, 53). These findings support the notion that cognitive impairment cannot be taken as a risk factor for aggression in schizophrenia (54, 55).

Our data join the few studies that examined auditory attentional performance in schizophrenia (20, 56). For example, Fresán et al. (53) failed to show a difference in attentional and inhibitory mechanisms between the forensic and non-forensic schizophrenia, using auditory ERP measures. The authors suggested that disruptive information sensory gating in schizophrenia leads to a sensory input overload. This sensory overload may explain difficulties in selective-attention to one speech channel.

### Integration of Channels and Channel Dominance

Supporting our third research prediction, non-forensic PwS' ratings did not indicate a bias for prosodic over semantic information (prosodic dominance), a hallmark of emotional processing in neurotypical populations. Instead, non-forensic PwS provided higher relative weightings for the semantic information than provided by forensic PwS and by controls. This impaired integration of information from two channels may relate to evidence on deficits in multisensory integration, common in schizophrenia (57–59). The lack of prosodic dominance for non-forensic PwS is in contrast with our previous findings on a preserved prosodic dominance for forensic PwS [similar to their matched controls (23)]. It is also notable that differences between processing of positive and negative emotions, as indicated for the forensic group, were not replicated in the current study with the non-forensic group. Instead, non-forensic PwS in our study were found to be impaired in emotional integration across all three tested emotions.

Taken together with our findings on emotional identification and discrimination, it appears that forensic and non-forensic schizophrenia differ in emotional processing but not in selective-attention. The findings suggest that difficulties in spoken-emotions processing are not necessarily associated with schizophrenia (34). Whereas forensic PwS have a relatively preserved (or at most marginally impaired) spoken emotional processing, non-forensic PwS are highly impaired in this ability. This may relate to a group difference in motivational deficit, a core negative symptom of schizophrenia, with severe functional outcomes (60, 61). The term motivational deficit relates to reduced goal-directed behavior and associated internal processes (e.g., drive) that lead individuals to actively initiate and perform tasks (62, 63). Severe motivational deficits in non-forensic schizophrenia may further impair emotional processing specifically when the task is demanding, such as when asked to integrate emotional channels in the current study [general rating task; for the cost of integration, see (64, 65)]. However, with relatively increased motivation, forensic PwS may be able to overcome some schizophrenia-related deficits and integrate emotional channels similarly to matched controls. Indeed, the literature suggests that negative symptoms are less characteristic of forensic schizophrenia than of general schizophrenia (66, 67). As negative symptoms are strongly associated with neurological abnormalities, it is feasible that forensic PwS may be less compromised neurologically than non-forensic PwS (67). Indeed, our results resonate with a recent notion that violent forensic PwS are best characterized by emotional and interpersonal aspects rather than by specific neuropsychological dysfunctions (37).

### Caveats and Future Studies

The study has several limitations. The clinical sample recruited for the non-forensic PwS group included only males. This was done to better compare to the male-only group of forensic PwS and controls in our previous study (23). Future studies may wish to expand this examination of emotional processing to female non-forensic PwS to assess possible gender differences. Note, non-forensic PwS in our study are all community dwellers, possibly suggesting a lower severity of schizophrenia than observed for the forensic group. However, this may serve to further support our results, as the non-forensic group was more impaired in emotional processing than the forensic group. Furthermore, although the study does not include a diagnostic measure to compare groups on cognitive abilities, all three groups were matched on (years of) education, a reliable gauge for linguistic skills (68, 69). However, it is still possible that group-differences on the T-RES may partially relate to basic cognitive abilities [e.g., working memory (70)]. Further studies are encouraged to test this possible link.

The T-RES paradigm by itself has a few limitations, such as the use of a single professional female actress to produce all stimuli, and the assessment of basic simple emotions [for a full list of limitations associated with the T-RES, see (27)]. The latter may serve to amplify the importance of our findings as former studies suggested that when using basic emotions, emotional processing was preserved for individuals with autism spectrum disorders without intellectual disabilities (26, 49). Future studies may wish to examine processing of complex emotions in forensic and non-forensic schizophrenia.

### Clinical Implications

The current findings have clinical implications, as some treatment programs for PwS target difficulties in emotional processing to improve social interactions and emotion dysregulation (71–73). The literature shows that for PwS, clinical symptoms may be modified through specific cognitive and behavior approaches, such as emotion regulation strategies. For example, emotion processing forms a key target in the Integrated Neurocognitive Therapy for schizophrenia patients (INT) (74). Several emotional targets are included in INT, such as identification and definition of basic emotions and their prototypical characteristics, affect recognition training, as well as emotion regulation training. The T-RES paradigm in that sense may be used first as a gauge for emotional processing, assessing progress along the treatment. Indeed, the current data further support the use of the T-RES as a sensitive tool in differentiating clinical populations in their processing of spoken emotions. As a response to COVID-19 social restrictions, a remote adaptation (an online version) of the T-RES has been validated (iT-RES) (26). This makes the clinical application of the tool more feasible. The T-RES may also be adapted as a practice tool, forming an “affective exercise,” where patients can train on specific emotional processing tasks. Finally, as forensic, and non-forensic PwS were found to be differentiated based on T-RES performance, this and other emotional processing tests may be added to the arsenal of diagnostic tools used in clinical assessment of schizophrenia.

The current study also has clear clinical implications for forensic PwS. The enhanced processing of spoken emotions indicated for forensic over non-forensic PwS may be an important (but not sufficient) part of their violent behavior. Indeed, the literature suggests that using the emotional understanding of others to inform behavior (“mastery”) is essential for the extreme kind of violent crimes committed by forensic PwS in Maximum Secure Units (31, 75). Structuring specialized intervention programs targeted at reducing (or even preventing) violence and recidivism in this population may rely on these preserved abilities and target emotional regulation [for a discussion, see (76)]. However, as noted by Bo et al. (37), violent PwS constitute a heterogeneous population with multiple etiologies. Thus, treatment programs in this population must consider symptomatology, traits and history (31, 32, 39, 77), as well as sensitivity to specific emotional categories (23, 76, 78), while strengthening social-emotional skills.

## Data Availability Statement

The raw data supporting the conclusions of this article will be made available by the authors, without undue reservation.

## Ethics Statement

The studies involving human participants were reviewed and approved by Bar-Ilan University IRB. The patients/participants provided their written informed consent to participate in this study.

## Author Contributions

Research was designed and conducted under the supervision of RL and BB-D. Data analysis was conducted by BB-D and MI. All authors written the manuscript.

## Funding

BB-D's lab was partially supported by a grant from the Israeli Science Foundation (ISF).

## Conflict of Interest

The authors declare that the research was conducted in the absence of any commercial or financial relationships that could be construed as a potential conflict of interest.

## Publisher's Note

All claims expressed in this article are solely those of the authors and do not necessarily represent those of their affiliated organizations, or those of the publisher, the editors and the reviewers. Any product that may be evaluated in this article, or claim that may be made by its manufacturer, is not guaranteed or endorsed by the publisher.
