# Supplementary material for: Processing of Spoken Emotions in Schizophrenia: Forensic and Non-forensic Patients Differ in Emotional Identification and Integration but Not in Selective Attention
Source: Front Psychiatry. 2022 Mar 21;13:847455. doi: 10.3389/fpsyt.2022.847455 (PMC8977511; doi:10.3389/fpsyt.2022.847455)

Appendix A. The full MLM analyses, comparing non-forensic PwS recruited for the current study, with controls taken from Leshem et al [21].

Emotional Identification		Selective-Attention		Integration: Prosodic-Dominance	
Intercept	*F*(1,67.2) = 3174.66, *p* < .001 .	Intercept	*F*(1,68.3) = 3703.10 , *p* < .001	Intercept	*F*(1,64.8) = 2854.63, *p* < .001
Group	*F*(1,64.1) = 0.32 , *p* = .57	Group	*F*(1,63.0) = 27.99, *p* < .001	Group	*F*(1,63.0) = 0.97, *p* = .33
Native-Language	*F*(1,63.0) = 0.36, *p* = .55	Native-Language	*F*(1,63.0) = 0.16, *p* = .69	Native-Language	*F*(1,63.0) = 1.11, *p* = .30
Target-Emotion	*F*(2,65.0) = 9.46, *p* < .001	Target-Emotion	*F*(2,65.0) = 4.9 , *p* = .01	Target-Emotion	*F*(2,65.0) = 41.10, *p* < .001
Target-Channel	*F*(1,66.5) = 3.33, *p* = .07	Target-Channel	*F*(1,66.8) = 0.06, *p* = .80		
Emotional-Identification	*F*(1,66.1) = 249.4, *p* < .001	Selective-Attention	*F*(1,69.0) = 9.97, *p* = .002	Prosodic-dominance	*F*(1,64.8) = 14.06, *p* < .001
Emotional-Identification * Native-Language	*F*(1,63.0) = 6.69, *p* = .012	Selective-Attention* Native-Language	*F*(1,63.0) = 0.69, *p* = .41	Prosodic-dominance* Native Language	*F*(1,63.0) = 7.50, *p* = .008
Emotional-Identification* Target-Emotion	*F*(2,65.0) = 11.98, *p* < .001	Selective-Attention* Target-Emotion	*F*(2,65.0) = 1.6, *p* = .21	Prosodic-dominance* Target-Emotion	*F*(2,65.0) = 32.49, *p* < .001
Emotional-Identification* Target-Channel	*F*(1,65.7) = 1.73, *p* = .19	Selective-Attention* Target-Channel	*F*(1,65.9) = 4.66, *p* = .035		
Emotional-Identification* Group	*F*(1,63.8) = 44.7, *p* < .001	Selective-Attention* Group*	*F*(1,65.1) = 5.65, *p* = .02	Prosodic-dominance* Group*	*F*(1,63.0) = 12.94, *p* = .001
Emotional-Identification* Group* Target-Channel	*F*(2,64) = 2.95, *p* = .06	Selective-attention* Group* Target-Channel	*F*(2,64) = 3.03, *p* = .055		

Appendix B

A graphic description of difference scores in the T-RES tasks, separately for non-forensic PwS (black bars, data from the current study), forensic PwS (gray bars, taken from Leshem et al., 2020) and Controls (light gray bars, Leshem et al., 2020). All data are estimates of MLM models averaged across the three emotion rating blocks. The error bars represent standard errors. (A) Identification, comparing target emotion-present and target-emotion-absent trials, averaged across the prosodic- and semantic-rating tasks; (B) Selective-attention, comparing congruent and incongruent trails, in the semantic-rating task; (C) Prosodic Dominance, comparing prosodic and semantic trials.

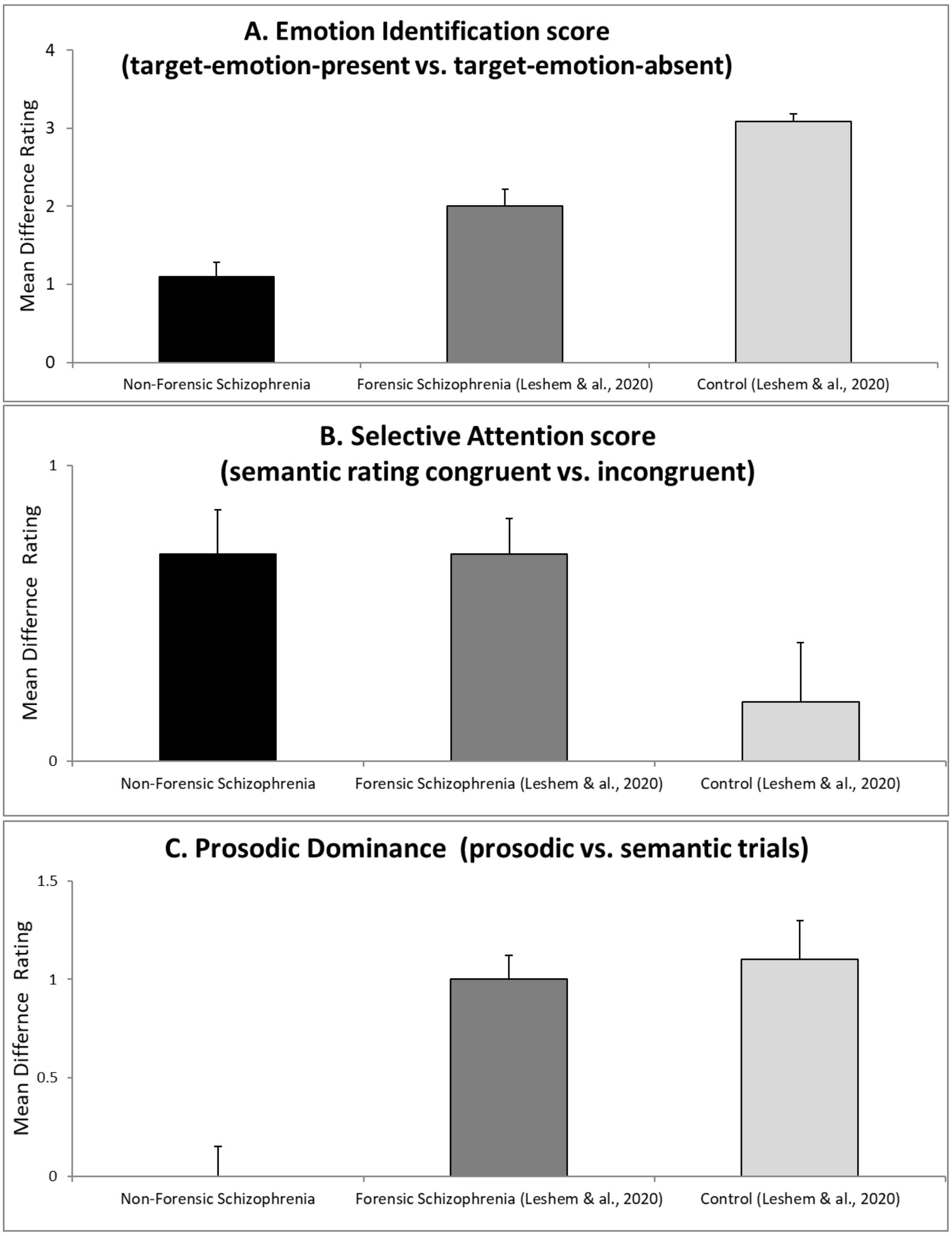

Supplement: Supplementary file 1 [file Data_Sheet_1.pdf]
